# Botulinum Neurotoxin-A Injection in Adult Cervical Dystonia and Spastic Paresis: Results From the INPUT (INjection Practice, Usage and Training) Survey

**DOI:** 10.3389/fneur.2020.570671

**Published:** 2020-09-16

**Authors:** Tae Mo Chung, Luis Jorge Jacinto, Carlo Colosimo, Kailash P. Bhatia, Julie Tiley, Roongroj Bhidayasiri

**Affiliations:** ^1^Faculty of Medicine, Institute of Physical Medicine and Rehabilitation, University of São Paulo, São Paulo, Brazil; ^2^Serviço de Reabilitação de adultos, Centro de Medicina de Reabilitacao do Alcoitão, Alcabideche, Portugal; ^3^Department of Neurology, Azienda Ospedaliera S.Maria, Terni, Italy; ^4^Institute of Neurology, University College London, London, United Kingdom; ^5^Global Medical Affairs, Ipsen, Boulogne Billancourt, France; ^6^Chulalongkorn Centre of Excellence for Parkinson's Disease & Related Disorders, Department of Medicine, Faculty of Medicine, Chulalongkorn University and King Chulalongkorn Memorial Hospital, Thai Red Cross Society, Bangkok, Thailand

**Keywords:** Botulinum toxin-A, cervical dystonia, patient management, spastic paresis, rehabilitation, continuous medical education (CME), practices

## Abstract

Botulinum toxin-A (BoNT-A) is an effective treatment for cervical dystonia (CD) and spastic paresis (SP), but it requires in-depth knowledge of anatomy and injection techniques. The Ixcellence Network® is an educational programme to provide neurology, neuropaediatrics, and physical medicine and rehabilitation (PMR) specialists with access to best clinical practices and innovations regarding SP and CD management with BoNT-A. To assess the benefits of such educational programmes and identify unmet needs, a multidisciplinary scientific committee designed INPUT (INjection Practice, Usage & Training), an international multicentric survey describing training and practices among this trained and experienced population. A self-completed questionnaire was sent online to 553 trainees and 14 trainers from the Ixcellence Network®. Among the 131 respondents, 92% specialized in PMR (48%) or neurology (44%), with a mean experience of 15.5 years in their clinical fields and 10.9 years of BoNT-A injection. Most of them (98%) reported having received training before performing their first BoNT-A injection and attending specific courses on how to perform it without any instrumental guidance (76%), and with ultrasound (73%), electrical stimulation (44%) or electromyography (41%). In terms of practices, 92% of respondents reported using at least one guidance technique while injecting, with ultrasound being the most used technique (48%). Attending specific courses was significantly associated with greater self-confidence and use, e.g. for injection with ultrasound, mean self-confidence, on a scale from 1 (not confident) to 10 (fully confident), was 7.9 for trained respondents (vs 4.0 for untrained respondents, *p* < *0.001*) of whom 70% stated that they used this technique regularly or systematically (vs. 11% of untrained healthcare professionals (HCPs), *p* < *0.0001*). Moreover, 84% of respondents reported having trained colleagues, residents or fellows through theoretical (70%) or practical teaching in individuals (80%) or in small groups (65%). Overall, 86% of respondents reported a notable increase over the past 5 years of the number of patients treated with BoNT-A. INPUT is the first international survey describing training and practices in SP and CD management of physicians who attended a dedicated educational programme. The results highlighted the importance of training for self-confidence, and the use of specific techniques and new approaches.

## Introduction

Botulinum toxin-A (BoNT-A), a neurotoxin produced by an anaerobic bacterium *Clostridium botulinum*, is a well-established and effective treatment for a number of neurological movement disorders associated with muscle hyperactivity, including adult spastic paresis (SP) from stroke, multiple sclerosis, traumatic brain or spinal cord injury, pediatric SP from cerebral palsy and adult cervical dystonia (CD) (1–6).

BoNT-A is administered by local injections into the muscles involved in the movement disorder. Its effectiveness depends on several factors including the injector's skills and knowledge, since accurate muscle selection, injection site selection and adequate drug dosage are key to achieving the best results (7–10). Proper clinical examination is required, as SP and CD present a wide diversity of patterns (11, 12). The localization technique can also affect BoNT-A outcomes. Palpation or instrumentally guided injection with ultrasound (US), electrical stimulation (ES) or electromyography (EMG) are commonly used to identify targeted muscles for injection in SP and CD treatment (12). Use of these targeting techniques is very much dependent on injector's prior training, confidence and the availability of equipment.

BoNT-A treatment should be tailored individually to each patient. When selecting which muscles to inject, the disease pattern, as well as patient and carer needs, should be taken into account to optimize management and outcomes (9, 13, 14). A pre-treatment evaluation and discussion with both the patient and the carer is essential to set optimal individual therapeutic goals. This step allows treatment efficacy to be measured and the strategy to be reassessed at each injection cycle to plan further treatment (14, 15).

Optimal BoNT-A treatment requires not only specific training in SP and CD management, but also in-depth knowledge of muscle anatomy and BoNT-A injection techniques, which are skills acquired only with time and commitment (9, 15, 16). However, there is no widely accepted standardized training for BoNT-A treatment. Although there are guidelines describing the diagnosis and treatment of SP and CD, there are no clear recommendations on how to perform the injections in different patients and conditions (16, 17). Physicians looking for experience and additional expertise on BoNT-A treatment are usually mentored by their peers under variable degrees of supervision (1). This may explain the wide variety of practices observed, with healthcare professionals (HCPs) choosing their approach to muscle selection, injection technique and follow-up therapy according to their preference, experience, confidence, and access (8, 18).

A steering committee of six international experts in SP and CD management developed the Ixcellence Network®, an international educational programme to provide physicians specialized in SP and CD with an opportunity to access best clinical practices and innovations concerning treatment with BoNT-A (19). Ten training centers were selected by the steering committee based on their expertise (five on SP, three on CD and two on pediatric SP). The training approach combines advanced theoretical courses and practical training, and addresses innovative methods and concepts concerning patient diagnosis, clinical and instrumental evaluation, muscle identification, goal setting, tailored treatment administration, and rehabilitation methods (e.g. Goal Attainment Scaling (GAS), Gait analysis, Col-Cap concept, guidance techniques, etc.). HCPs (the vast majority of whom were medical practitioners) participating in these courses were selected based on their experience in the management of adult and/or pediatric SP and/or CD, with at least 2 years of practical experience with regular botulinum toxin clinics.

The INjection Practice, Usage & Training (INPUT) survey was designed to describe training, practices regarding patient management with BoNT-A and how they evolved in this trained and experienced population, with the aims of evaluating the benefits of training and identifying unmet needs.

## Methods

The INPUT survey is an international, observational and multicentric study conducted among practitioners specialized in neurology, neuropaediatrics, or physical medicine and rehabilitation (PMR), and trained through the Ixcellence Network® programme.

### Survey Design

A multidisciplinary scientific committee designed a self-completed questionnaire including 41 items divided into two sections: a general part with 19 questions on experience, training and clinic organization, and a specific part customized for each of the three indications (SP: 9 questions, pediatric SP: 10 questions, CD: 9 questions). The respondents were asked to answer a minimum of 28 (e.g., HCPs exclusively managing adults with SP) to a maximum of 41 questions (HCPs treating all three indications). The questionnaire included multiple-choice and open-ended questions. None of the questions was compulsory.

### Recipients

Between 1 June 2018 and 26 February 2019, an online version of the questionnaire was sent by email to 553 HCPs managing patients with SP and/or CD, who attended at least one Ixcellence Network^®^ training session between 2012 and 2018, and 14 programme trainers. Three reminders were sent on a weekly basis. Recipients were instructed to answer the online questionnaire anonymously, without opening their patient files or looking at their activity statistics.

### Data Collection

The following parameters were collected: (1) general information*:* country of practice, experience in their field and in BoNT-A injection, training on different approaches/techniques, confidence in using these approaches, trainer activity; (2) clinic organization: time dedicated to BoNT-A injection, number of injectors in the department, waiting time for appointments, usage of guidance techniques for BoNT-A injection; (3) patient profile and management: type of patients treated and specific information related to each indication (e.g., main challenges for BoNT-A injection, time dedicated to discussion with patients, frequency of setting up a rehabilitation programme in combination with BoNT-A injections, etc.); (4) how their clinical practice evolved over the past 5 years (Supplementary Figure 1).

For patient profile and management results, only adult SP and CD data were reported in this publication, which focuses on adult SP and CD management.

### Data Analysis

Data were analyzed using the Statistical Analysis Software SAS®. Results were described using numbers and percentages for qualitative variables, and means and standard deviations for quantitative variables. Continuous data were analyzed using a Student's *t*-test (following a Student's t distribution) and categorical data using Fisher's exact test (based on a hypergeometric distribution). The analysis was completed with subgroup analysis according to respondents' specialty (neurology vs. PMR), experience in the field (<15 years vs. ≥ 15years), experience in BoNT-A injection (<10 years vs. ≥10 years) and time allocated to BoNT-A injections (one half day vs. > one half day per week).

## Results

Among the 553 trainees and 14 trainers who received the questionnaire, 131 HCPs answered the survey (i.e., response rate of 23.1%).

### Respondents' Profile (Table 1)

Respondents represented 38 countries, located in Europe (60%), Latin America (25%), Africa/Middle East (12%), Asia/Oceania (3%), and specialized in PMR (48%), neurology (44%), neuropediatrics (5%), orthopedics (2%) or other specialties (1%). The mean duration of respondents' experience was 15.5 years in their clinical field (range 1–41 years) and 10.9 years of BoNT-A injection experience (range 1–29 years).

**Table 1:** Characteristics of respondents.

Area of practice (*n* = 131)	Europe	60%
	Latin America	25%
	Africa/Middle East	12%
	Asia/Oceania	3%
Medical speciality (*n* = 131)	Physical medicine and rehabilitation	48%
	Neurology	44%
	Neuropediatrics	5%
	Orthopedics	2%
	Other: Clinical neurophysiology	1%
Experience in their field (*n* = 125)	Range	1–41 years
	Mean ± sd	15.5 ± 8.7 years
Experience in BoNT-A injection (*n* = 129)	Range	1–29 years
	Mean ± sd	10.9 ± 6.2 years

### Type of Patients Treated by Respondents

Respondents reported managing adult SP (72%), CD (48%), and pediatric SP (38%). Indications treated were significantly different according to speciality, with a higher number of PMR specialists managing SP (adult: 86 vs. 64% for neurologists, *p* = *0.006*; pediatric: 59 vs. 7% for neurologists, *p* < *0.0001*), and a higher number of neurologists treating CD (91 vs. 14% for PMR specialists, *p* < *0.0001*).

### Clinic Organization

Respondents reported seeing monthly, on average, 23 ± 20 adult outpatients with SP for consultation and 13 ± 11 for BoNT-A injection; 28 ± 38 children outpatients with SP for consultation and 11 ± 14 for BoNT-A injection; 12 ± 18 CD outpatients for consultation and 17 ± 34 for BoNT-A injection. The mean number of injectors in the respondents' department was 2.9 ± 1.6 (range 1–10). On average, respondents allocated 1.8 ± 1.2 half-days per week to BoNT-A injection. New patients requiring BoNT-A injection waited an average of 5.7 ± 5.3 weeks (range 1–30 weeks) to get their first appointment with the HCPs. The planning of the next injection's session was predominantly flexible, according to the patient's needs (57% of respondents), with an average waiting time of 19.1 ± 23.5 days (range 0–100 days*)* and predominantly fixed for 43% of respondents, with an average interval of 12.3 ± 5.4 weeks (range 1–24 weeks).

### Training (Table 2)

**Table 2:** Training in different approaches and techniques during medical residency.

		**Trained HCPs *n* = 131, %**	**Subgroup analysis**		***P*-value Fischer's Exact test**
			**Trained PMR specialists (*n* = 64)**	**Trained neurologists (*n* = 55)**	
Assessment and treatment objectives	Goal Setting	73%	80%	62%	0.042
	Scales	62%	64%	56%	0.454
	Cop-Cap concept	51%	17%	29%	<0.0001
	Gait analysis	42%	61%	34%	0.006
BoNT-A injection	Anatomical landmarks/palpation without instrumental guidance	76%	75%	78%	0.829
	Ultrasound-guided injection	73%	28%	56%	0.003
	Electrostimulation-guided injection	44%	48%	38%	0.273
	Electromyography-guided injection	41%	72%	73%	0.999
Rehabilitation	Rehabilitation in adult SP	57%	81%	40%	<0.0001
	Self-rehabilitation in adult SP	33%	37%	33%	0.701
	Rehabilitation in CD	24%	17%	33%	0.057
	Self-rehabilitation in CD	15%	6%	27%	0.002

#### Patient's Assessment and Treatment Objectives

Seventy three percentage of respondents attended courses dedicated to goal setting, 62% on scales, 51% on the Col-Cap concept and 42% on GAIT analysis, with significant differences between specialties: more PMR specialists were trained on goal setting and GAIT analysis compared to neurologists (80 vs. 62%, *p* = *0.0418* and 61 vs. 34%, *p* = *0.0057*, respectively), while more neurologists were trained on the Col-Cap concept (69 vs. 17% for PMR specialists, *p* > *0.0001*). On average, respondents attended 2.3 out the 4 courses dedicated to assessment and treatment objectives (2.3 for PMR specialists and 2.2 for neurologists, NS).

#### BoNT-A Injection

Almost all respondents (98%) reported that they were trained before performing their first BoNT-A injection. This initial training was done under a colleague tutelage (77%), practical sessions (65%) and theoretical courses (65%) with 42% of respondents receiving these three types of initial training. During their medical residency, most of the respondents were trained on how to perform the injection without any instrumental guidance (76%) with US (73%), with ES (44%) or with EMG (41%). Neurologists were significantly more trained in using EMG than PMR specialists (56 vs. 28%, respectively, *p* = *0.003*). Regarding guidance technique usage, on average, respondents were trained on 1.6 out the 3 topics (1.5 for PMR specialists and 1.7 for neurologists, NS).

#### Rehabilitation Approaches

Respondents reported specific training sessions on rehabilitation for adult SP (57%), self-rehabilitation for adult SP (33%), rehabilitation for CD (24%) and self-rehabilitation for CD (15%). Subgroup analysis showed a higher number of PMR specialists trained in rehabilitation for adult SP compared with neurologists (81 vs. 40%, *p* < *0.0001*). Contrarily, more neurologists were trained in self-rehabilitation for CD (27 vs. 6%, *p* < *0.0023*). On average, respondents attended training on 1.4 out the three topics related to rehabilitation (1.5 for PMR specialists and 0.8 for neurologists, NS), and on 0.6 out the three topics related to self-rehabilitation (0.6 for PMR specialists and neurologists, NS).

#### Training of Colleagues

Among the respondents, 84% stated that they had opportunities to train some colleagues, residents or fellows through at least one of the following formats: individual practical teaching (80%), theoretical teaching (70%) or practical teaching in small groups (65%).

### BoNT-A Injection in Clinical Practice

#### With Palpation Only

BoNT-A injection was performed without using anything except palpation and anatomical references, regularly or systematically (48%), scarcely (35%), and never (17%), without any difference between PMR specialists and neurologists.

#### With Localization Devices (Table 3)

Almost all respondents (92%) reported using at least one guidance device scarcely, regularly or systematically to inject BoNT-A. US was the most used with 48% of respondents using it regularly or systematically vs. 40% for ES and 41% for EMG. Most of the respondents (64%) declared using at least two devices and 30% used all three localization devices. There was no significant difference between specialties, but some trends were observed, for example, ES was used by more PMR specialists while EMG seemed to be predominately used by neurologists.

**Table 3:** Guidance technique usage for BoNT-A injection.

	**Overall (*n* = 123)**	**PMR specialists (*n* = 60)**	**Neurologists (*n* = 50)**
None	8%	7%	6%
US only	15%	13%	16%
EMG only	8%	7%	8%
ES only	4%	8%	0%
EMG + ES	9%	7%	14%
EMG + US	10%	7%	16%
ES + US	15%	28%	2%
EMG + ES + US	30%	23%	38%

### SP Patient Management

**Figure 1:**
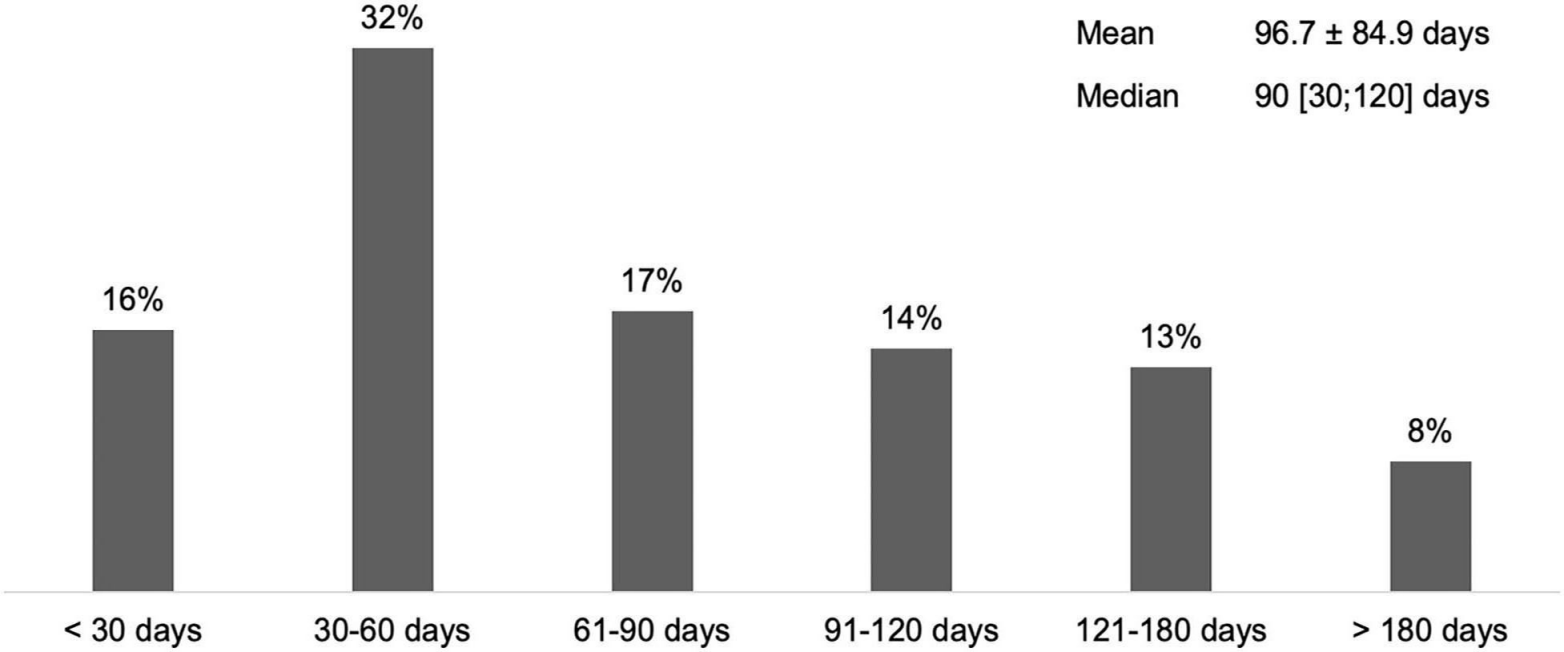
Average time between stroke and first treatment with BoNT-A in adult SP (*n* = 90).

According to 65% of respondents who manage adults with SP (*n* = 94), the average time to first BoNT-A treatment after stroke was <90 days (Figure 1).

The main drivers for prescribing BoNT-A were to improve active function (79%), quality of life (66%) and passive function (58%), whereas muscle selection (69%), dose determination (48%), and injection point selection (36%) were considered challenging by the respondents (Figure 3A).

Among respondents treating adult SP (*n* = 89), 94% reported using localization devices at least scarcely (22% only one type, 35% 2 types, 37% all 3 types). US was the most used guidance device with 51% of respondents using it regularly or systematically vs. 46% for ES and 40% for EMG (Table 3).

Almost all respondents (98%) reported discussing treatment goals with their patient or the carer/family at least once every two or three consultations (Figure 4A) and 65% reported combining systematically BoNT-A treatment with a rehabilitation programme (Figure 5A).

### CD Patient Management

**Figure 2:**
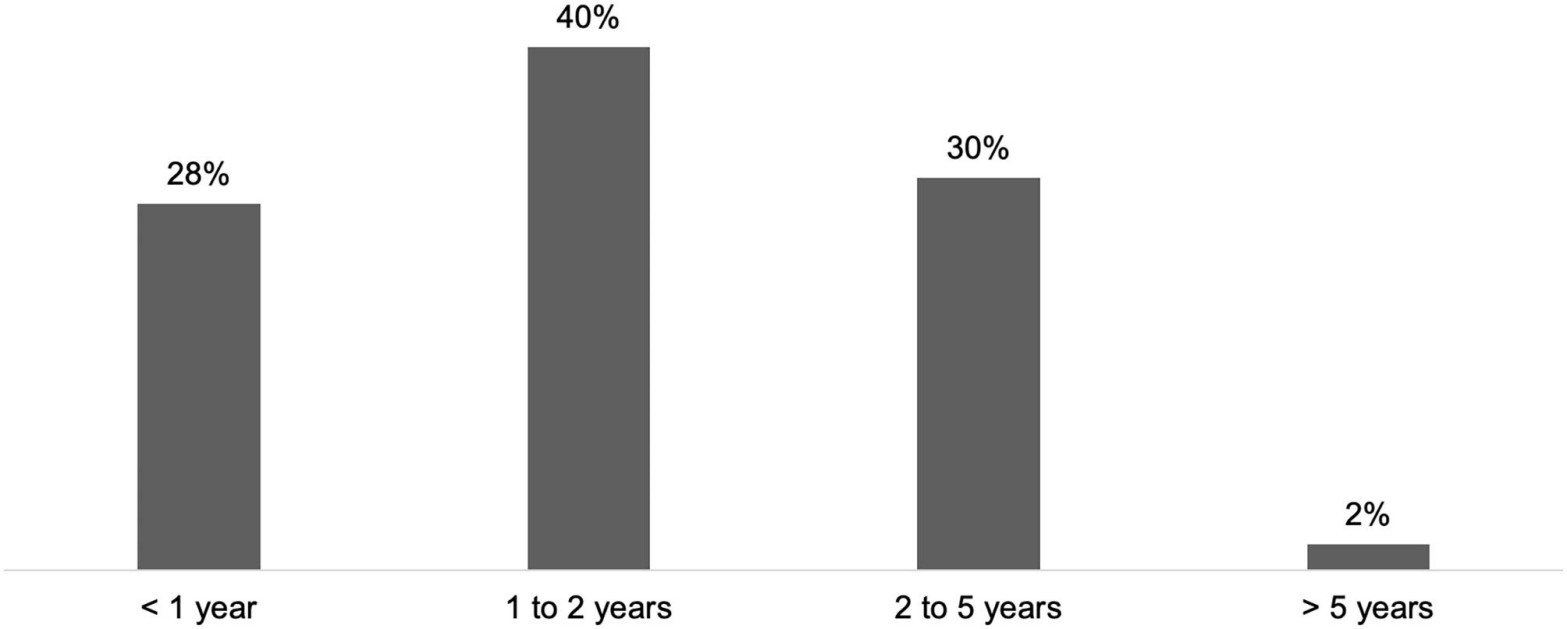
Average time between symptom onset and first consultation for CD patients (*n* = 60).

In the practice of HCPs managing CD (*n* = 68), the first consultation occurred during the first year after symptom onset for 28% of respondents, and between 1 and 2 years for 40% of respondents (Figure 2). According to 32% of respondents, the average time was higher than 2 years (2 to 5 years: 30%; > 5 years: 2%).

**Figure 3:**
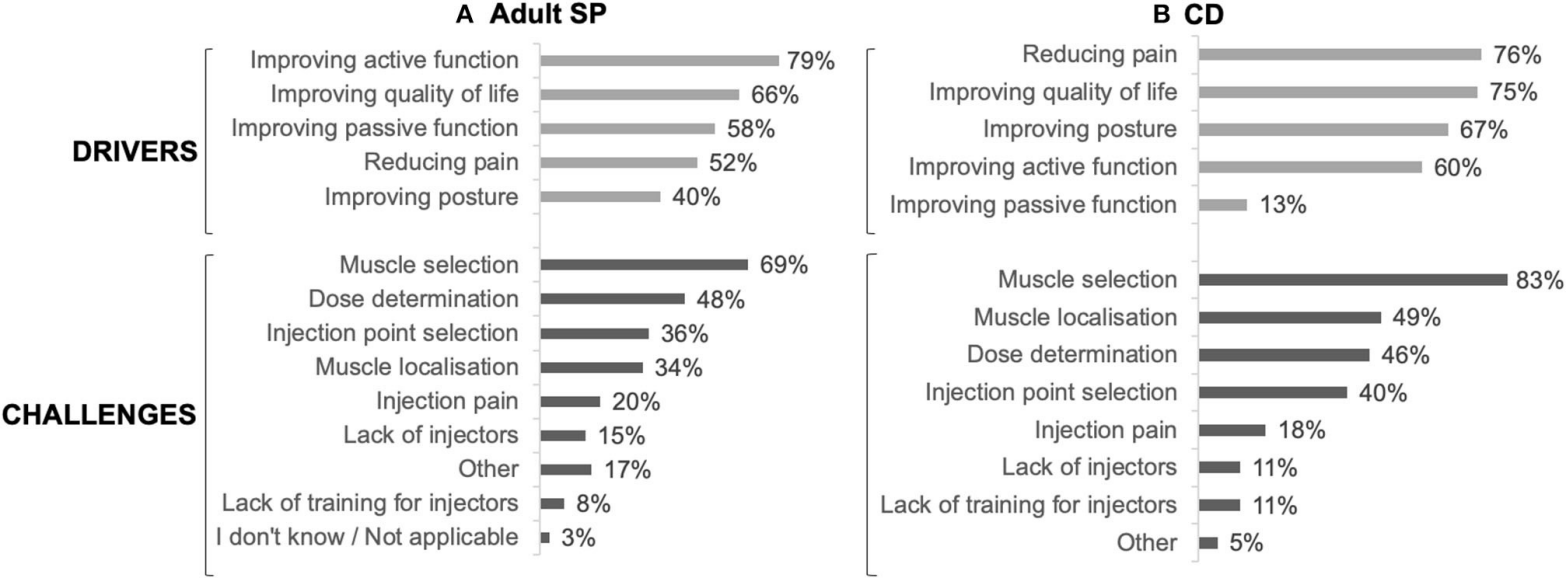
Drivers and challenges for BoNT-A injection in adult SP **(A)** (*n* = 94) and CD **(B)** (*n* = 63).

In CD patients, the main drivers for BoNT-A treatment were reducing pain (76%), improving quality of life (75%) and improving posture (67%), whereas muscle selection (83%), muscle localization (49%) and dose determination (46%) were considered challenging (Figure 3B).

Subgroup analysis showed that 92% of respondents managing CD (*n* = 60) performed BoNT-A with the help of a guidance technique at least scarcely (25% used only one type, 30% 2 types, 37% all three types). EMG was the most used localization device with 50% of respondents using it regularly or systematically vs. 42% for US and 37% for ES (Table 3).

**Figure 4:**
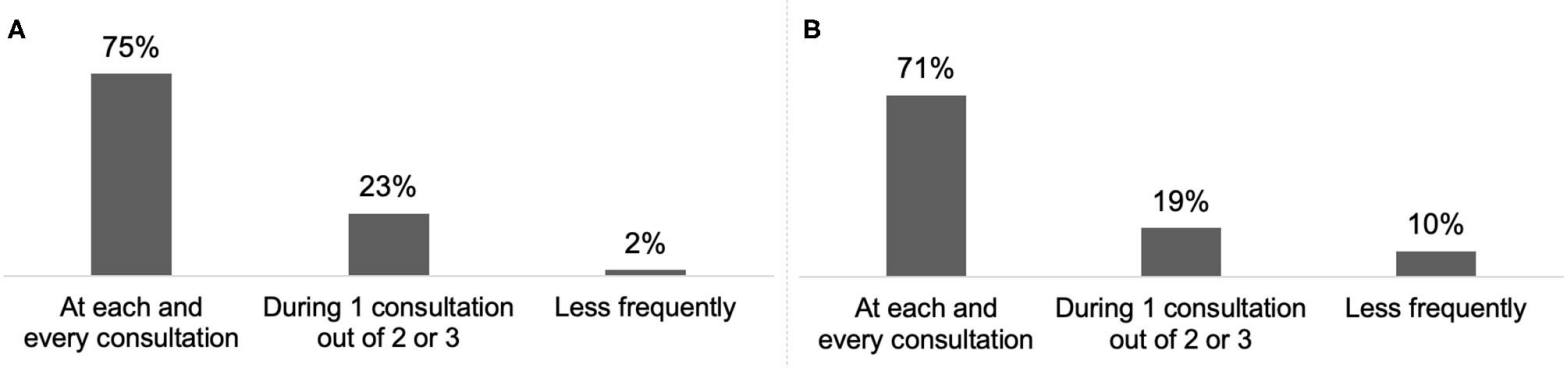
Frequency of goal setting in adult SP **(A)** (*n* = 92) and CD **(B)** (*n* = 62).

**Figure 5:**
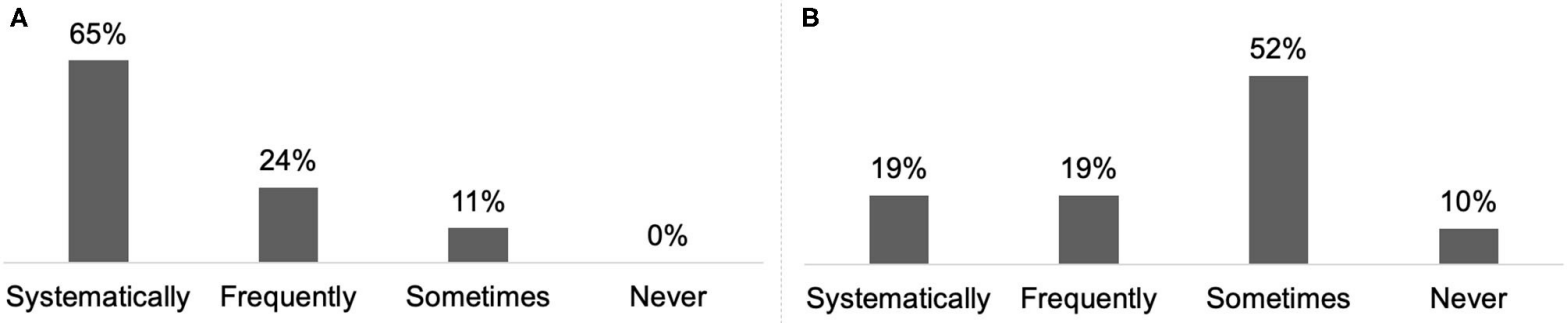
Frequency of rehabilitation prescription in adult SP **(A)** (*n* = 91) and CD **(B)** (*n* = 63).

For 70% of respondents, treatment goals were discussed with CD patients and their carer/family at each consultation (Figure 4B) and 19% reported combining systematically BoNT-A treatment with a rehabilitation programme (Figure 5B).

### Impact of Training on Self-Confidence (Table 4)

Self-confidence in using specific approaches or techniques was measured on a scale from 1 (not confident) to 10 (fully confident). Comparing the scores amongst the HCPs who declared having been trained vs. the ones who did not allowed an estimation of the influence of training on self-confidence. Data reported in Table 4 highlighted a significant positive effect of training. Overall, HCPs who followed specific training on goal setting, injection using instrumental guidance and rehabilitation approaches reported significantly greater self-confidence compared to those who did not.

**Table 4:** Impact of training on self-confidence.

			Self-confidence*	
		***n***	**Mean score ± sd (*n*)**	***P*-value *t*-test**
Goal setting	Trained	93	7.8 ± 1.8	0.001
	Not trained	28	6.3 ± 2.7	
Scales	Trained	80	8.0 ± 1.6	<0.001
	Not trained	39	5.7 ± 2.2	
Col-Cap concept	Trained	52	7.8 ± 1.9	<0.001
	Not trained	37	3.6 ± 2.8	
Gait analyses	Trained	65	7.0 ± 2.3	<0.001
	Not trained	39	4.7 ± 2.4	
Ultrasound-guided injection	Trained	92	7.9 ± 2.5	<0.001
	Not trained	15	4.0 ± 2.9	
Electromyography-guided injection	Trained	54	8.0 ± 2.1	<0.001
	Not trained	37	4.5 ± 2.9	
Electrostimulation-guided injection	Trained	55	8.4 ± 1.7	<0.001
	Not trained	37	5.6 ± 3.3	
Rehabilitation in adult SP	Trained	75	8.3 ± 1.7	<0.001
	Not trained	26	4.8 ± 3.3	
Self-rehabilitation in adult SP	Trained	42	7.6 ± 1.7	<0.001
	Not trained	46	5.1 ± 3.1	
Rehabilitation in CD	Trained	31	7.1 ± 2.2	<0.001
	Not trained	39	4.6 ± 2.7	
Self-rehabilitation in CD	Trained	18	6.9 ± 1.7	<0.001
	Not trained	43	4.1 ± 2.6	

* *Self-confidence was rated on a scale from 1 = not confident to 10 = fully confident*.

### Impact of Training on Practice (Table 5)

To assess the impact of training on practice, the frequency of the different approaches and techniques was compared between respondents who followed dedicated courses and those who did not. Regarding goal setting, no significant difference was observed in term of usage/prescription between specifically trained or untrained HCPs. Contrarily, the use of guidance technique was significantly higher among HCPs who received specific training: US, EMG and ES were used regularly or systematically by 70, 55, and 54% of trained HCPs, respectively, vs. 11% (*p* < *0.0001*), 15% (*p* = *0.0015*), and 21% (*p* = *0.0001*) of untrained HCPs.

**Table 5:** Impact of training on usage of different approaches.

			Usage of the different approaches*	
		***n***	**Percentage of respondents**	***P*-value Exact Fisher test**
Goal setting in adult SP	Trained	70	76	0.783
	Not trained	22	73%	
Goal setting in CD	Trained	42	72	0.999
	Not trained	20	70%	
Ultrasound-guided injection	Trained	90	70	<0.0001
	Not trained	18	11%	
Electromyography-guided injection	Trained	53	55%	0.001
	Not trained	48	15%	
Electrostimulation-guided injection	Trained	54	54%	0.0001
	Not trained	43	21%	
Rehabilitation in adult SP	Trained	68	91	0.266
	Not trained	23	82%	
Rehabilitation in CD	Trained	25	60%	0.003
	Not trained	34	21%	

* *At every consultation for goal setting; regularly or systematically for BoNT-A injection; systematically or frequently for rehabilitation*.

The frequency of rehabilitation approaches did not differ among respondents managing adult SP who attended dedicated courses and those who did not. However, training had a significant positive impact on respondents prescribing rehabilitation in CD with 60% of trained HCPs vs. 21% of untrained ones combining rehabilitation with BoNT-A treatment systematically (*p* = *0.0028*).

### Evolution of Practice Over the Five Past Years

The majority of respondents (86%) reported a notable increase of the number of patients treated with BoNT-A over the past 5 years. Regarding the technique employed, while most did not report a change in BoNT-A injection with palpation only, or with EMG or with ES, 83% stated that their usage of US increased over the five past years. Accordingly, increase in the usage of goal setting, scales, Col-Cap concept and GAIT analysis was reported by 70, 51, 73, and 53% of respondents, respectively. Rehabilitation approaches are also more frequently included in patient management with a reported increase in the prescription of physiotherapy in combination with BoNT-A treatment (53%) and of self-rehabilitation programmes (65%).

## Discussion

The aim of the INPUT survey was to describe training and practices of former Ixcellence Network® attendees in order to assess the impact of the programme and identify points for improvement.

Survey participants were representative of the Ixcellence Network® trainees. Among the 728 HCPs who attended courses between 2012 and 2017, 86% were PMR and neurology specialists, and 72% came from European countries (20). Most of the respondents were specialized in PMR and neurology (92%), and from Europe (60%). In addition, the number of respondents per indication was consistent with the number of dedicated courses proposed in Ixcellence Network® (five for adult SP, two for pediatric SP and three for CD). Respondent characteristics also confirmed that the attendee profiles matched the programme selection criteria. HCPs had to have at least 2 years of experience in treating adult SP, pediatric SP or CD with BoNT-A to attend an Ixcellence Network® training course. Respondents reported an extensive experience in both their field and BoNT-A injection, with an average practice of over 10 years.

The Ixcellence Network® programme targets specialized practitioners, keen to develop further their expertise with training and innovative approaches. Results from the INPUT survey confirmed that this population is well-trained, but remains keen to improve further their knowledge and skills. Almost all respondents received training before performing BoNT-A injection alone and half of them by different methods (theoretical courses, practical sessions and colleague tutelage). Most of them also completed their medical residency with dedicated training on specific approaches and techniques covering patient assessment, treatment goals, BoNT-A treatment and rehabilitation approaches. Interestingly, the most attended courses were goal setting, BoNT-A injection without instrumental guidance or with US (three of every four HCPs), while less attended courses included BoNT-A injection with EMG or ES and rehabilitation approaches.

Regarding daily practice, respondents were well aware of innovative approaches and applied them frequently. Almost all HCPs used at least one guidance device and one in every three HCPs used all three devices (US, EMG and ES). Published data indicate improved accuracy of injection with instrumental guidance for SP and CD, compared with palpation only (21–23). For both SP and CD, goals were discussed at every consultation by around three quarters of HCPs. This step is essential to define achievable, realistic and relevant goals with the patient (1). Results from the ULIS-II study, a large international observational cohort study of real-life practice and outcomes in post-stroke treatment of upper limb spasticity with BoNT-A, showed that 80% of patients achieved their previously set treatment goals using Goal Attainment Scaling (GAS), mainly in terms of passive and active functions, thus improving their daily life (18). Combination of BoNT-A treatment with rehabilitation and/or self-rehabilitation, whose benefits were shown in both SP and CD treatment, have been reported to be included in patient management (24–26). BoNT-A treatment after stroke within the first 3 months was frequent among respondents (63%). This approach is supported by recent studies that show that early intervention (within the first 12 weeks) may modify the disease course and delay the presentation of symptoms (27, 28). In the literature, the average delay for CD diagnosis varies from 44 months to 6.8 years and is associated with a negative impact on quality of life (29, 30). Within the INPUT survey population, this delay appeared to be <2 years.

The proportion of PMR specialists and neurologists was well-balanced in the sample, which allowed comparison of both groups. Subgroup analysis revealed significant differences in terms of type of patients treated, and training and trends regarding the BoNT-A injection technique. PMR specialists, who mostly manage SP, are more likely to be trained in goal setting, GAIT analysis (used for both SP diagnosis and assessment) and rehabilitation in SP. This group mostly uses US and ES for BoNT-A injection. Contrarily, neurologists mostly manage CD and focus their training on the Col-Cap concept (used for CD diagnosis and assessment), in BoNT-A injection with EMG and, more recently, US.

The INPUT survey also highlighted interesting differences between SP and CD management. Although most of the respondents reported discussing goals with patients and carers/family for both indications in at least one in every three visits, only 2% of HCPs treating SP did so less frequently vs. 10% of HCPs treating CD. Rehabilitation is also more frequently prescribed for SP patients compared to CD patients. Treatment goals for BoNT-A injection also differed, with pain reduction being more important for CD than SP. This is consistent with the results from an international survey of 1,071 CD patients showing that pain is frequent in this population (reported by 66% of respondents) and its reduction is highly expected (62% of respondents) (31).

In our study, attending specific courses was always significantly associated with greater self-confidence. However, the impact of training on practice differed depending on the approach or technique. Trained HCPs performed BoNT-A injection with a localization device and prescribed rehabilitation for CD more regularly than untrained respondents, but training had no significant impact on goal setting usage or rehabilitation prescription for adult SP. Attending dedicated courses seemed more important for usage of techniques requiring specific skills and knowledge, or new approaches. In a recent survey conducted by the American Academy of Neurology (AAN) among graduating adult and child neurology residents in the USA, two of three respondents reported a desire for additional residency training in BoNT-A injections (32).

Although attendee satisfaction and changes in self-confidence and practices are evaluated within the programme immediately after each course and 6 months later, the aim of this study was to better evaluate the overall programme impact after 5 years. Results confirmed that the programme has a positive impact on the former attendees. Respondents reported changing their practices regarding BoNT-A treatment over the past 5 years with more patients treated and a growing use of US. Patient assessment and treatment goal-setting approaches were also more frequently used, especially goal setting, Col-Cap concept and rehabilitation, which are more recently introduced approaches. Interestingly, BoNT-A injection with US (three of four HCPs) was among the most attended training courses, while less popular courses were BoNT-A injection with EMG or ES and new rehabilitation approaches. This confirmed the development of these approaches in recent years. Moreover, 84% of the INPUT survey respondents reported teaching others, which is consistent with the data collected within the programme. A total of 82% of trainees reported having shared the information learned during the courses with colleagues/peers and 89% said they would consider training their colleagues (20). This suggests that, through the different courses offered, Ixcellence Network® influences a great number of physicians.

The results of the INPUT survey confirmed that Ixcellence Network® attendees are well-trained and motivated specialists. However, some skills can be optimized, e.g., goal setting in CD, adjunct therapies to BoNT-A. New centers may be opened in the future to provide dedicated courses on these developing topics.

Before injecting BoNT-A for the first time, many of the respondents learned from peers. It is important to set up specific courses for HCPs who are not specialized in the field and to develop a standard for national/international programmes.

There are limitations in the survey methodology of this study. Firstly, the response rate was relatively low (23%), which highlights the difficulties in organizing surveys among specialist physicians who are a professional group with low survey response rates in general (32). In our study, the response rate may be influenced by a number of factors, such as the number of items in the questionnaire and the lag period between the training and the survey invitation. Although consistent with similar surveys found in the literature (RR usually around 20–30%) (33) this makes the extrapolation of our results to real life only approximate. Nevertheless, to the best of our knowledge, INPUT is the first international survey characterizing a clinical training programme in order to assess its impact and identify improvement opportunities. The few survey results published describe general or local practices or a specific aspect of management (33–38). Respondents are probably the most motivated to undertake training and innovative approaches, and the most involved in the Ixcellence Network® programme. A direct link cannot be established between the Ixcellence Network® and the impact of training on self-confidence and practice changes because, for example, respondents may have attended courses outside of the programme. The questionnaire design did not allow comparison between practices in terms of BoNT-A treatment for SP and CD. The questions on BoNT-A injection technique were included only in the general question section. Since a significant number of respondents reported treating both indications, it was not possible to statistically compare both subpopulations.

## Conclusion

INPUT is the first international survey describing the training and practices in SP and CD management by physicians who attended a dedicated training programme. The results highlighted the importance of training for self-confidence, and the use of specific techniques and new approaches. The Ixcellence Network® may have a positive impact on the daily practice of physicians already using BoNT-A, which is consistent with its aim to upgrade the skills of specialized and experienced HCPs. This study also helped identify areas for improvements, such as the opening of new centers dedicated to emerging approaches in SP and CD management.

## Data Availability Statement

The raw data supporting the conclusions of this article will be made available by the authors, without undue reservation.

## Ethics Statement

Ethical review and approval was not required for the study on human participants in accordance with the local legislation and institutional requirements. Written informed consent for participation was not required for this study in accordance with the national legislation and the institutional requirements.

## Author Contributions

All authors developed the concept and the methodology of this study, including the design of the questionnaire, under the supervision of RB. Data analysis and writing of the original draft were conducted by TC and RB. JT, CC, KB, and LJ reviewed, edited, and approved the manuscript.

## Conflict of Interest

The training courses and the development of this manuscript were supported by Ipsen Pharma. Ixcellence Network^®^ is a registered trademark owned by Ipsen Pharma. All authors except JT received honoraria, and a speaker and consultancy fee from Ipsen Pharma. TC has not personally received any research funding and has no financial interest in BoNT and received honoraria from Ipsen Pharma as a speaker at symposia, training courses and participation in the advisory board, and has participated in several clinical studies sponsored by Ipsen Pharma. LJ received research grants and honoraria as a scientific advisor, lecturer and peer trainer from Allergan and Merz. CC received grant support as a speaker and advisory board committee member from Ipsen Pharma, Merz Pharma, Zambon Pharma, Sunovion Pharmaceuticals, and Bial. KB received personal compensation for activities with UCB Pharma and from Novartis as a speaker. JT is an employee of Ipsen Pharma. RB received honoraria from Boehringer Ingelheim, GlaxoSmithKline, Abbott, Novartis, Roche, Lundbeck Pharmaceuticals, and grants from the Thailand Research Fund, a Ratchadapiseksompoj faculty grant from Chulalongkorn University, a research grant from the Neurological Society of Thailand, a research unit grant from Chulalongkorn University, a Parkinson's Disease Center Development Grant from the Thai Red Cross Society, the Newton Fund (UK) and intellectual property rights from the Laser cane for Parkinson disease, Electronic Parkinson's disease diary and Parkinson's glove.

## Supplementary Material

Supplementary Figure 1 INPUT survey questionnaire.
