# Supplementary material for: Botulinum Neurotoxin-A Injection in Adult Cervical Dystonia and Spastic Paresis: Results From the INPUT (INjection Practice, Usage and Training) Survey
Source: Front Neurol. 2020 Sep 16;11:570671. doi: 10.3389/fneur.2020.570671 (PMC7525121; doi:10.3389/fneur.2020.570671)
Supplement: Supplementary Figure 1 — INPUT survey questionnaire. [file Data_Sheet_1.PDF]

iXCELLENCE® NETWORK
iN-Research

INPUT
INjection Practice, Usage & Training survey

GENERAL INFORMATION

1. **What is your country of practice?**

1. **What is your specialty?** *(check all that apply)*

- ☐ Neurology
- ☐ Neuropaediatrics
- ☐ Physical Medicine and Rehabilitation (PMR)
- ☐ Orthopaedics
- ☐ Other, please specify:

2. **For how many years have you been practicing in this field?**
   years

3. **For how many years have you been injecting BoNT-A?**
   years

4. **Before performing alone your first BoNT-A injection on your patients, how were you trained?** *(check all that apply)*

- ☐ Theoretical courses
- ☐ Practical sessions
- ☐ Injection under a colleague tutelage
- ☐ None
- ☐ I don't know / Non applicable

5. **Have you ever attended any specific training on the following approaches since the beginning of your medical residency?** *(check all that apply)*

ASSESSMENT & TREATMENT OBJECTIVES

Goal Setting	☐
Scales	☐
Col-cap concept (Cervical Dystonia)	☐
Gait analysis	☐

BONT-A INJECTION GUIDANCE TECHNIQUES

Anatomical landmarks / palpation	☐
Electromyography (EMG)	☐
Electrostimulation (ES)	☐
Ultrasound (US)	☐

**BONT-A INJECTION ON CADAVER**	☐

REHABILITATION

Rehabilitation in Adult Spastic Paresis	☐
Self-rehabilitation in Adult Spastic Paresis	☐
Rehabilitation in Pediatric Spastic Paresis	☐
Self-rehabilitation in Pediatric Spastic Paresis	☐
Rehabilitation in Adult Cervical Dystonia	☐
Self-rehabilitation in Adult Cervical Dystonia	☐

6. **How confident are you in using the following approaches?**
(on a scale from 1 to 10, with 1 = not confident and 10= fully confident)

ASSESSMENT & TREATMENT OBJECTIVES

Goal Setting	1 ☐ 2☐ 3 ☐ 4☐ 5 ☐ 6☐ 7☐ 8☐ 9 ☐ 10☐
Scales	1 ☐ 2☐ 3 ☐ 4☐ 5 ☐ 6☐ 7☐ 8☐ 9 ☐ 10☐
Col-cap concept (Cervical Dystonia)	1 ☐ 2☐ 3 ☐ 4☐ 5 ☐ 6☐ 7☐ 8☐ 9 ☐ 10☐
Gait analysis	1 ☐ 2☐ 3 ☐ 4☐ 5 ☐ 6☐ 7☐ 8☐ 9 ☐ 10☐

BONT-A INJECTION GUIDANCE TECHNIQUES

Anatomical landmarks / palpation	1 ☐ 2☐ 3 ☐ 4☐ 5 ☐ 6☐ 7☐ 8☐ 9 ☐ 10☐
Electromyography (EMG)	1 ☐ 2☐ 3 ☐ 4☐ 5 ☐ 6☐ 7☐ 8☐ 9 ☐ 10☐
Electrostimulation (ES)	1 ☐ 2☐ 3 ☐ 4☐ 5 ☐ 6☐ 7☐ 8☐ 9 ☐ 10☐
Ultrasound (US)	1 ☐ 2☐ 3 ☐ 4☐ 5 ☐ 6☐ 7☐ 8☐ 9 ☐ 10☐

REHABILITATION

Rehabilitation in Adult Spastic Paresis	1 ☐ 2☐ 3 ☐ 4☐ 5 ☐ 6☐ 7☐ 8☐ 9 ☐ 10☐
Self-rehabilitation in Adult Spastic Paresis	1 ☐ 2☐ 3 ☐ 4☐ 5 ☐ 6☐ 7☐ 8☐ 9 ☐ 10☐
Rehabilitation in Pediatric Spastic Paresis	1 ☐ 2☐ 3 ☐ 4☐ 5 ☐ 6☐ 7☐ 8☐ 9 ☐ 10☐
Self-rehabilitation in Pediatric Spastic Paresis	1 ☐ 2☐ 3 ☐ 4☐ 5 ☐ 6☐ 7☐ 8☐ 9 ☐ 10☐
Rehabilitation in Adult Cervical Dystonia	1 ☐ 2☐ 3 ☐ 4☐ 5 ☐ 6☐ 7☐ 8☐ 9 ☐ 10☐
Self-rehabilitation in Adult Cervical Dystonia	1 ☐ 2☐ 3 ☐ 4☐ 5 ☐ 6☐ 7☐ 8☐ 9 ☐ 10☐

7. **Did you have the opportunity to train some of your colleagues/residents/fellows on the following approaches?** ☐ Yes ☐ No ☐ I don't know / Non applicable

   If yes,..

   On which approach: *(Check all that apply)*

   ☐ Assessment & treatment objectives
   ☐ BoNT-A injection guidance techniques
   ☐ Rehabilitation
   ☐ None of the above

   Through which format: *(Check all that apply)*

   ☐ Theoretical teaching
   ☐ Practical teaching in small groups (<10)
   ☐ Individual practical teaching
   ☐ None of the above

CLINIC ORGANIZATION

8. **How many half-days per week do you allocate to BoNT-A injections?**
   half-days/week

9. **How many BoNT-A injectors are there in your department (including yourself)?**
   injectors

10. **For a new patient, what is the average time to get an appointment with you for BoNT-A injection?** weeks

11. **How are the next injections planned afterwards?** *(choose one of the following answers)*

    ☐ Predominantly fixed schedule (at fixed intervals)
    → On average at what frequency are the injection planned? weeks

    ☐ Predominantly flexible schedule (according to the patient's need)
    → What is the average time to get an appointment once asked for? days

12. **Do you reconstitute the BoNT-A product yourself?** ☐ Yes ☐ No ☐ I don't know / Non applicable

13. **How often do you perform BoNT-A injection <u>without guidance</u> (thanks to palpation only)?** *(choose one of the following answers)*

Systematically	On a regular basis	Scarcely	Never	I don't know / Non applicable
☐	☐	☐	☐	☐

14. **How often do you perform BoNT-A injection using the following guidance techniques?** *(choose one of the following answers)*

	Systematically	On a regular basis	Scarcely	Never, I do not have the material	Never, despite having the material	I don't know / Non applicable
Electromyography	☐	☐	☐	☐	☐	☐
Electrostimulation	☐	☐	☐	☐	☐	☐
Ultrasound	☐	☐	☐	☐	☐	☐

PATIENT PROFILE & MANAGEMENT

15. **What type of patients do you inject with BoNT-A on a regular basis?** *(check all that apply)*

☐ Adults with Spastic Paresis (if ticked → please answer to the questions 15 to 25)

☐ Children with Spastic Paresis (if ticked → please answer to the questions 26 to 35)

☐ Adults with Cervical Dystonia (if ticked → please answer to the questions 36 to 45)

ADULT SPASTIC PARESIS

16. **On average, per month, how many adult outpatients with spastic paresis do you see:**
for clinical consultation? /month
for BoNT-A injection? /month

17. **For your adult patients with spastic paresis, what are the 3 main DRIVERS for BoNT-A injection?** *(Please select at most 3 answers)*

- ☐ Improving active function
- ☐ Improving passive function
- ☐ Improving posture
- ☐ Improving quality of life
- ☐ Reducing pain
- ☐ Other(s)
- ☐ I don’t know / Non applicable

18. **For your adult patients with spastic paresis, what are the 3 main CHALLENGES for BoNT-A injection?** *(Please select at most 3 answers)*

- ☐ Muscle selection
- ☐ Muscle localisation
- ☐ Injection point(s) selection
- ☐ Dose determination
- ☐ Injection pain
- ☐ Lack of injectors
- ☐ Lack of training for injectors
- ☐ Other(s)
- ☐ I don’t know / Non applicable

19. **In your centre, what is the average time to first post-stroke treatment with BoNT-A?**
days

20. **How often do you discuss treatment goals with your adult patients with spastic paresis, the family / caregiver?**

- ☐ At each and every consultation
- ☐ During 1 consultation out of 2 or 3
- ☐ Less frequently
- ☐ I don’t know / Non applicable

21. **What is the average time dedicated to this discussion?**
☐ < 5 min ☐ > 5 min ☐ I don’t know / Non applicable

22. **While discussing goals with your adult patients with spastic paresis, which of the following tools do you use?** *(multiple answers possible)*

- ☐ Specific GAS (Goal Attainment Scale)
- ☐ Other scale(s), please specify
- ☐ Auto-questionnaire, please specify
- ☐ Other(s) tool(s), please specify
- ☐ None
- ☐ I don’t know / Non applicable

23. **For your adult patients with spastic paresis, how often is a rehabilitation program combined with the BoNT-A treatment?**

☐ Systematically ☐ Frequently ☐ Sometimes ☐ Never ☐ I don’t know / Non applicable

24. **In your practice, what is the percentage of adult patients with spastic paresis that benefits from the following type of rehabilitation combined with BoNT-A treatment?**

Total must be 100%

Physiotherapy alone	Physiotherapy + self-rehabilitation	None
out of 100	out of 100	out of 100

CHILDREN SPASTIC PARESIS

25. **On average, per month, how many paediatric outpatients do you see:**
for clinical consultation? /month
for BoNT-A injection? /month

26. **On average for your paediatric patients with spastic paresis, from what age do you start the BoNT-A treatment?**

2	3	4	5	6	7	8	9	10	11	12	13	14	15	16
☐	☐	☐	☐	☐	☐	☐	☐	☐	☐	☐	☐	☐	☐	☐

☐ I don’t know / Non applicable

27. **When injecting, which method(s) do you use to avoid pain?**
- ☐ Local anaesthesia, e.g. cold spray, anaesthetic cream
- ☐ Inhalation anaesthetic
- ☐ General anaesthesia
- ☐ Distraction
- ☐ Other(s)
- ☐ I don’t know / Non applicable

28. **For your paediatric patients with spastic paresis, what are the 3 main DRIVERS for BoNT-A injection?** *(multiple answers possible)*
- ☐ Improving active function
- ☐ Improving passive function
- ☐ Improving posture
- ☐ Delay surgery
- ☐ Improving quality of life
- ☐ Reducing pain
- ☐ Other(s)
- ☐ I don’t know / Non applicable

29. **For your paediatric patients with spastic paresis, what are the 3 main CHALLENGES for BoNT-A injection?** *(multiple answers possible)*
- ☐ Muscle selection
- ☐ Muscle localisation
- ☐ Injection point(s) selection
- ☐ Dose determination
- ☐ Injection pain
- ☐ Lack of injectors
- ☐ Lack of training for injectors
- ☐ Parents reluctance
- ☐ Other(s)
- ☐ I don’t know / Non applicable

30. **How often do you discuss treatment goals with your paediatric patients with spastic paresis, the family / caregiver?**

- ☐ At each and every consultation
- ☐ During 1 consultation out of 2 or 3
- ☐ Less frequently
- ☐ I don't know / Non applicable

31. **What is the average time dedicated to this discussion?**

☐ < 5 min ☐ > 5 min ☐ I don't know / Non applicable

32. **While discussing goals with your paediatric patients with spastic paresis, which of the following tools do you use?**

- ☐ Specific GAS (Goal Attainment Scale)
- ☐ Other scale(s), please specify
- ☐ Auto-questionnaire, please specify
- ☐ Other(s) tool(s), please specify
- ☐ None
- ☐ I don't know / Non applicable

33. **For your paediatric patients with spastic paresis, how often is a rehabilitation program combined with the BoNT-A treatment?**

☐ Systematically ☐ Frequently ☐ Sometimes ☐ Never ☐ I don't know / Non applicable

34. **In your practice, what is the percentage of paediatric patients with spastic paresis that benefits from the following type of rehabilitation combined with BoNT-A treatment?**

Total must be 100%

Physiotherapy alone	Physiotherapy + self-rehabilitation	None
out of 100	out of 100	out of 100

35. **In your practice for adult patients with cervical dystonia, what is the average time between symptom onset and first consultation?**

< 1 year	1 to 2 years	2 to 5 years	> 5 years	I don't know / Non applicable
☐	☐	☐	☐	☐

36. **On average, per month, how many adult outpatients with cervical dystonia do you see:**
for clinical consultation? /month
for BoNT-A injection? /month

37. **For your adult patients with cervical dystonia, what are the 3 main DRIVERS for BoNT-A injection?** *(multiple answers possible)*
- ☐ Improving active function
- ☐ Improving passive function
- ☐ Improving posture
- ☐ Improving quality of life
- ☐ Reducing pain
- ☐ Other(s)
- ☐ I don't know / Non applicable

38. **For your adult patients with cervical dystonia, what are the 3 main CHALLENGES for BoNT-A injection?** *(multiple answers possible)*
- ☐ Muscle selection
- ☐ Muscle localisation
- ☐ Injection point(s) selection
- ☐ Dose determination
- ☐ Injection pain
- ☐ Lack of injectors
- ☐ Lack of training for injectors
- ☐ Other(s)
- ☐ I don't know / Non applicable

39. **How often do you discuss treatment goals with your adult patients with cervical dystonia, the family / caregiver?**
- ☐ At each and every consultation
- ☐ During 1 consultation out of 2 or 3
- ☐ Less frequently
- ☐ I don't know / Non applicable

40. **What is the average time dedicated to this discussion?**
☐ < 5 min ☐ > 5 min ☐ I don't know / Non applicable

41. **While discussing goals, which of the following tools do you use?** *(multiple answers possible)*
- ☐ Scale(s), please specify
- ☐ Auto-questionnaire, please specify
- ☐ Other(s) tool(s), please specify
- ☐ None
- ☐ I don't know / Non applicable

42. **How often is a rehabilitation program combined with the BoNT-A treatment?**
☐ Systematically ☐ Frequently ☐ Sometimes ☐ Never ☐ I don't know / Non applicable

43. **In your practice, what is the percentage of adult patients with cervical dystonia that benefits from the following type of rehabilitation combined with BoNT-A treatment?**

Total must be 100%

Physiotherapy alone	Physiotherapy + self-rehabilitation	None
out of 100	out of 100	out of 100

EVOLUTION OF YOUR PRACTICE OVER THE 5 PAST YEARS

Acccording to your practice, which one of the following statements is true regarding:

44. **The number of your patients treated with BoNT-A?**

- ☐ I treat LESS patients with BoNT-A than 5 years ago
- ☐ My practice has NOT CHANGED over the past 5 years
- ☐ I treat MORE patients with BoNT-A than 5 years ago
- ☐ I don't know / Non applicable

45. **The average time to get an appointment for BoNT-A injection with you?**

- ☐ The average time to get an appointment is SHORTER than 5 years ago
- ☐ The average time to get an appointment has NOT CHANGED over the past 5 years
- ☐ The average time to get an appointment is LONGER than 5 years ago
- ☐ I don't know / Non applicable

46. **The following approaches?**

	I use / prescribe it MORE often than 5 years ago	I use / prescribe it LESS often than 5 years ago	My practice has NOT CHANGED over the past 5 years	I don't know / Non applicable
**ASSESSMENT & TREATMENT OBJECTIVE**				
Goal Setting	☐	☐	☐	☐
Scales	☐	☐	☐	☐
Col-cap concept (Cervical Dystonia)	☐	☐	☐	☐
Gait analysis	☐	☐	☐	☐

BONT-A INJECTION GUIDANCE TECHNIQUES

Anatomical landmarks	☐	☐	☐	☐
Electromyography (EMG)	☐	☐	☐	☐
Electrostimulation (ES)	☐	☐	☐	☐
Ultrasound (US)	☐	☐	☐	☐

REHABILITATION

Physiotherapy associated with BoNT-A	☐	☐	☐	☐
Self-rehabilitation associated with BoNT-A	☐	☐	☐	☐
